# *Ehrlichia chaffeensis* TRP47 enters the nucleus via a MYND-binding domain-dependent mechanism and predominantly binds enhancers of host genes associated with signal transduction, cytoskeletal organization, and immune response

**DOI:** 10.1371/journal.pone.0205983

**Published:** 2018-11-08

**Authors:** Clayton E. Kibler, Sarah L. Milligan, Tierra R. Farris, Bing Zhu, Shubhajit Mitra, Jere W. McBride

**Affiliations:** 1 Department of Pathology, University of Texas Medical Branch, Galveston, Texas, United States of America; 2 Department of Microbiology and Immunology, University of Texas Medical Branch, Galveston, Texas, United States of America; 3 Center for Biodefense and Emerging Infectious Diseases, University of Texas Medical Branch, Galveston, Texas, United States of America; 4 Sealy Center for Vaccine Development, University of Texas Medical Branch, Galveston, Texas, United States of America; 5 Institute for Human Infections and Immunity, University of Texas Medical Branch, Galveston, Texas, United States of America; State University of New York Upstate Medical University, UNITED STATES

## Abstract

*Ehrlichia chaffeensis* is an obligately intracellular bacterium that establishes infection in mononuclear phagocytes through largely undefined reprogramming strategies including modulation of host gene transcription. In this study, we demonstrate that the *E*. *chaffeensis* effector TRP47 enters the host cell nucleus and binds regulatory regions of host genes relevant to infection. TRP47 was observed in the nucleus of *E*. *chaffeensis*-infected host cells, and nuclear localization was dependent on a variant MYND-binding domain. An electrophoretic mobility shift assay (EMSA) demonstrated that TRP47 directly binds host DNA via its tandem repeat domain. Utilizing chromatin immunoprecipitation followed by high-throughput DNA sequencing (ChIP-seq) with *E*. *chaffeensis*-infected cells, TRP47 was found to bind at multiple sites in the human genome (n = 2,051 at *p* < 10^−30^). Ontology analysis identified genes involved in functions such as immune response, cytoskeletal organization, and signal transduction. TRP47-bound genes included RNA-coding genes, many of these linked to cell proliferation or apoptosis. Comparison of TRP47 binding sites with those of previously-identified *E*. *chaffeensis* nucleomodulins identified multiple genes and gene functional categories in common including intracellular transport, cell signaling, and transcriptional regulation. Further, motif analysis followed by EMSA with synthetic oligonucleotides containing discovered motifs revealed a conserved TRP47 DNA-binding motif. This study reveals that TRP47 is a nucleomodulin that enters the nucleus via a MYND-binding domain and appears to play a role in host cell reprogramming by regulation of transcription.

## Introduction

*Ehrlichia chaffeensis* is an obligately intracellular bacterium and the etiologic agent of human monocytic ehrlichiosis (HME), a life-threatening NIAID emerging tick-borne zoonosis. Symptoms of HME are nonspecific and include fever, headache, myalgia, and malaise. Progression can lead to a toxic shock-like syndrome resulting in hospitalization (about 50% of cases), and in some cases a more severe multisystem failure resulting in death (3% of cases) [1].

*E*. *chaffeensis* preferentially infects mononuclear phagocytes and subverts host defenses by direct activation of cellular pathways and through other complex molecular mechanisms that involve type 1-secreted ankyrin and tandem repeat protein (TRP) effectors. TRPs are immunoreactive proteins that contain protective linear antibody epitopes in the tandem repeat (TR) domains. In addition, previous studies have demonstrated that TRP effectors interact with a diverse array of host proteins involved in biological processes such as cell signaling, intracellular transport, metabolism, protein post-translational modification, apoptosis, and regulation of transcription [2]. Notably, *E*. *chaffeensis* also appears to target host gene transcription to manipulate apoptosis, cell differentiation, signal transduction, immune response, metabolism, and intracellular transport during infection [3,4]; however, the mechanisms by which *E*. *chaffeensis* alters transcription are not fully understood.

An increasing number of studies indicate that various pathogenic bacterial effectors can exert control over host cells by entering and acting within the nucleus [5]. Such effectors, also known as nucleomodulins, interact with histone or transcriptional regulator proteins, or even directly bind DNA to modify the host transcriptome. For example, transcription-activator-like (TAL) effectors of the plant pathogen *Xanthomonas* are translocated to the nucleus via canonical nuclear localization signals (NLS), where they directly bind host DNA via internal tandem repeat domains, and activate transcription of genes that promote infection [6,7].

Recently, *E*. *chaffeensis* effectors Ank200, TRP120, and TRP32 have been shown to function as nucleomodulins. Ank200 binds adenine-rich *Alu* elements in host promoter and intron regions, functional categories of identified target genes include transcriptional regulation, apoptosis, ATPase activity, and nuclear structure, and many of these genes are differentially expressed during infection [8]. TRP120 and TRP32 both bind host DNA at multiple genomic sites including gene promoters at a specific G-rich DNA motif via internal tandem repeat domains. Genes bound by TRP120 are implicated in cellular processes such as transcription regulation, apoptosis, and phosphorylation [9]. TRP32-bound genes are involved with immune cell activation, chromatin remodeling, and RNA transcription, and transcription of target genes is directly modulated by TRP32 [10]. The mechanism of nuclear translocation for ehrlichial effectors is unclear since they lack a canonical NLS, but likely involves an alternative mechanism such as post-translational modification and interaction with another protein that has an NLS. For instance, tyrosine phosphorylation of TRP32 is required for nuclear translocation [10].

*E*. *chaffeensis* TRP47, the most highly transcriptionally expressed ehrlichial gene during infection of mammalian cells, is known to interact with multiple host proteins that influence infection including proteins involved in transcriptional regulation and has been observed in the nucleus [11,12]. In this study, we determined that *E*. *chaffeensis* TRP47, similar to TRP120 and TRP32, translocates to the host cell nucleus, directly binds host DNA via its tandem repeat domain, and targets multiple genes involved in processes relevant to infection. Taken together, these results suggests that TRP47 acts as a nucleomodulin involved in an *E*. *chaffeensis* transcriptional reprogramming strategy.

## Materials and methods

### Cell culture and infection

*Ehrlichia chaffeensis* (Arkansas strain) was propagated in a human monocytic cell line (THP-1). THP-1 cells were maintained in RPMI 1640 (HyClone, Logan, UT) supplemented with 5% fetal bovine serum (FBS; HyClone), 2.05 mM L-glutamine, 1 mM sodium pyruvate, and 2.5 g/L glucose at 37°C in a 5% CO_2_ atmosphere. Human cervical epithelial adenocarcinoma cells (HeLa; ATCC, Manassas, Va.) for transfection were cultured in MEM (Hyclone) supplemented with 5% fetal bovine serum (FBS; HyClone).

### Antibodies

Polyclonal rabbit and mouse anti-TRP47 antibodies were generated against a peptide from the TRP47 tandem repeat region (ASVSEGDAVVNAVSQETPA) by a commercial vendor (Bio-Synthesis, Lewisville, TX). Pre-immune serum from the same rabbit was also used in this study as a control. Rabbit anti-Dsb antibody was produced as previously described [13].

### Immunofluorescence and confocal laser microscopy

TRP47 localization during infection was examined by immunofluorescence and confocal laser microscopy. Briefly, uninfected and *E*. *chaffeensis*-infected THP-1 cells were cytocentrifuged onto glass slides at 24, 48, and 72 h post infection and fixed in 4% paraformaldehyde in phosphate-buffered saline (Alfa Aeasar) at room temperature for 25 min. Cells were then permeabilized using 1% Triton X-100 with 2% bovine serum albumin (BSA; Sigma-Aldrich) in phosphate-buffered saline (PBS; Sigma-Aldrich) for 1 h, followed by incubation with 1:100 mouse anti-TRP47 and 1:100 rabbit anti-Dsb antibodies for 1 h. After incubation with primary antibodies, slides were washed 4 times in PBS and incubated with Alexa Fluor 488-conjugated goat anti-rabbit IgG (H+L) and Alexa Fluor 594-conjugated goat anti-rabbit IgG (H+L) secondary antibodies for 30 min. Finally, glass coverslips (Fisherbrand premium cover glass) were mounted using ProLong Gold antifade reagent with DAPI (4′,6-diamidino-2-phenylindole) (Cell Signaling Technology, Danvers, Mass.) and dried at room temperature overnight. Images were obtained using a Zeiss LSM 880 Airyscan super resolution laser scanning confocal microscope configured with an Axiovert inverted microscope with a c-Apochromat 40x/1.2 numerical aperture water immersion lens. UV laser (405 nm) and VIS laser (488 nm and 561 nm) were used to excite the sample and emissions were captured using band pass filters of 385 to 470nm (for DAPI), 505 to 530 nm (for Alexa Fluor 488 conjugate), and 560 to 615 nm (for Alexa Fluor 594 conjugate), respectively. Images were analyzed using Zenlite blue software, and z-stacks were constructed by imaging optical slices at 0.16 μm intervals.

### Expression constructs and site-directed mutagenesis

*E*. *chaffeensis* genomic DNA was extracted from infected THP-1 cells using the DNeasy Blood & Tissue Kit (QIAGEN, Hilden, Germany), and DNA concentration was determined with a NanoDrop ND-1000 spectrophotometer (Thermo Fisher Scientific). Oligonucleotide primers (S1 Table) were designed to amplify various regions of TRP47 for cloning into the pGEX-6P-1 N-terminal glutathione S-transferase (GST) fusion bacterial expression vector (GE Healthcare Life Sciences, Pittsburgh, Penn.) or the pAcGFP1-C InFusion Ready N-terminal Green Fluorescent Protein (GFP) fusion bacterial/mammalian expression vector (Clontech, Mountain View, Calif.). PCR products were generated from *E*. *chaffeensis* genomic DNA with HotMasterMix (5 PRIME, Gaithersburg, Md.) using the following thermal cycling program: 94°C for 2 min; 30 cycles of 94°C for 30 s, annealing temperature (5°C less than the lowest primer Tm) for 30 s, and 65°C for the appropriate extension time (30 s per 500 product base pairs); and 65°C for 7 min. Correct size was verified using the FlashGel DNA electrophoresis system (Lonza, Basel, Switzerland), and PCR products were purified with the MinElute PCR Purification Kit (Qiagen). PCR products for pGEX-6P-1 constructs were digested with EcoRI and SalI high-fidelity restriction enzymes (New England Biolabs, Ipswich, Mass.) and ligated into digested vector (Fast-Link DNA Ligase; Epicentre, Madison, Wis.). Constructs were transformed into TOP10 *E*. *coli* (Invitrogen, Carlsbad, Calif.), and transformants were selected by growth on LB agar with ampicillin. PCR products for pAcGFP1-C constructs were cloned into pre-linearized vector using the In-Fusion PCR Cloning Kit (Clontech), and transformants were selected by growth on LB agar with kanamycin. The DNA fragment encoding the His-tagged MYND-binding domain (His-MBD) was created by annealing primers flanking the region. A PCR product encoding TRP47 without the MYND-binding domain (No Motif construct) was amplified from a commercially synthesized pUc57 plasmid (GenScript) using the forward and reverse primers for the full-length GFP construct. PCR screening was used to verify correct insert size, and plasmids from positive colonies were isolated (QIAprep Spin Miniprep Kit, Qiagen) and sequenced to verify proper orientation and frame. Lysine-to-arginine mutation of the full-length TRP47 pAcGFP1-C construct was performed using the QuikChange II SiteDirected Mutagenesis Kit (Agilent, Santa Clara, Calif.) according to the manufacturer’s instructions. Mutating primers were created using the QuikChange Primer Design program. Plasmids from positive transformants were isolated and sequenced to verify presence of the correct mutation.

### Expression and purification of recombinant TRP47

Plasmids encoding full-length and tandem repeat region TRP47 GST-fusion constructs were transformed into TurboCells BL21(DE3) *E*. *coli* and plated on LB agar with ampicillin. Colonies were PCR-screened to verify presence of the insert, and positive transformants were regrown overnight in LB broth with ampicillin. Overnight cultures were diluted 1:20 in fresh growth medium and incubated with shaking at 37°C until an OD_600_ of about 0.5 was achieved. Protein expression was induced with 1 mM isopropyl β-D-1-thiogalactopyranoside (IPTG; Sigma-Aldrich) for 4 h, after which cultures were pelleted and stored at -80°C. Cell pellets were resuspended in Tris-HCl buffer and lysed by sonication in the presence of protease inhibitors (cOmplete mini, EDTA-free; Roche, Basel, Switzerland). Lysates were cleared by centrifugation at 12,000x*g* and 4°C for 30 min, and the supernatant was collected and incubated with Glutathione Sepharose 4B affinity resin (GE Healthcare Life Sciences) at 4°C with nutation overnight. After washing with Tris-HCl, bound protein was eluted from the resin with 50 mM glutathione. Dialysis was performed and the concentration measured using the Pierce BCA Protein Assay Kit (Thermo Fisher Scientific). Recombinant protein expression and purification were verified using SDS-PAGE and protein staining (AcquaStain Protein Gel Stain; Bulldog Bio, Portsmouth, N.H.).

### Ectopic expression of GFP-tagged TRP47 constructs

Plasmids for transfection (pAcGFP1-C FL, N, Ntrunc, TRC, C, MBD, No Motif, K49R, K71R, GFP control) were isolated from 150 mL overnight *E*. *coli* cultures with the PerfectPrep EndoFree Plasmid Maxi Kit (5 PRIME) and quantitated using a spectrophotometer. HeLa cells were seeded at a density of 0.8 x 10^5^ cells/mL in 8-well chamber slides 24 h prior to transfection. Transfections were performed in duplicate with Lipofectamine 2000 (Thermo Fisher Scientific) according to the manufacturer’s recommendations. Twenty-four hours after transfection, cells were fixed with acetone at -20°C for 10 min and mounted with ProLong Gold antifade reagent with DAPI. Slides were dried at room temperature overnight, viewed using an Olympus BX61 epifluorescence microscope, and analyzed with Slidebook software (version 5.0; Intelligent Imaging Innovations, Denver, CO).

### Chromatin immunoprecipitation and sequencing

*E*. *chaffeensis-*infected THP-1 cells harvested at 48 hpi were used for chromatin immunoprecipitation using the EZ Magna ChIP Kit (EMD Millipore). Briefly, cells were cross-linked using a final concentration of 1% formaldehyde for 10 min. Cells were then pelleted and lysed with lysis buffer and a dounce homogenizer. Lysate was sonicated on ice using Sonics Vibra-Cell (20 cycles, 30 s at 5 W output, 30 s rest) to generate chromatin fragments around 300 bp in length. To validate sheared chromatin samples and ChIP technique, ChIP was performed with anti-RNA polymerase II positive control antibody followed by measurement of fold enrichment of GAPDH nucleic acid by qPCR. TRP47 was immunoprecipitated using rabbit anti-TRP47 at optimized concentration and pre-immune serum was used as a control. Washing of IP reactions and reversal of crosslinks were performed according to the Magna ChIP protocol, and nucleic acid was purified and quantified using a Qubit fluorometer. Preparation of indexed DNA sample libraries involving PCR amplification with adapter ligation was performed by the UTMB Next Generation Sequencing Core using the NEBNext Ultra II DNA Library Kit. Library quality and quantity was evaluated using Agilent Bioanalyzer. Samples were sequenced using an Illumina HiSeq 1500 to generate about 40 million single-end 50 base pair sequence reads per sample. Sequence reads were analyzed by base calling and sequence quality filtering scripts with Illumina Pipeline software. Sequences were aligned to the human genome (UCSC hg38) using bowtie2. The output BAM files were processed by removing PCR duplicates, reads mapping to multiple genomic locations, and reads with low mapping scores. MACS [14] was then used to call peaks ranked by significance and BED files were generated. Hypergeometric Optimization of Motif EnRichment (HOMER) software [15] was also used to identify peaks for comparison, but downstream analyses focused on MACS peaks. Peaks were visualized using Integrated Genomics Viewer (IGV).

### Gene annotation and ontology

BED files consisting of highly significant TRP47 peaks were submitted to Genomic Regions Enrichment of Annotations Tool (GREAT) version 3.0 [16]. Peaks were associated with nearby genes using the Basal Plus Extension rule, which assigns genes a proximal regulatory region consisting of -5 kb to +1 kb from the transcription start site (TSS) and a distal regulatory region extending in both directions to the proximal regulatory region of any other genes but no more than 1000 kb. GREAT also utilizes a small set of curated regulatory domains from the literature. After the ChIP-seq peaks were assigned to nearby genes, the annotations from those genes were used to calculate enriched categories using region-based binomial and gene-based hypergeometric tests. The enriched ontology terms were filtered to include only those significant (*p <* 0.05) by both statistical tests. Significant TRP120 and TRP32 peaks were also submitted to GREAT to identify target genes and ontology terms in common between TRPs.

To associate TRP47 peaks with gene regulatory regions, the BEDTools *coverage* function was used to examine overlap of peaks with promoters from the Eukaryotic Promoter Database EPDnew (each TSS extended -5 kb to +1 kb using BEDTools *slop*) and enhancers from the GeneHancer database [17]. To determine significance of enrichment in different genomic regions, experimental peaks were randomly permuted throughout the genome (excluding centromere regions which are not well represented in whole-genome sequencing data) using BEDTools *shuffle* to generate 10 random peak sets [18]. These control peak sets were compared to the experimental peaks using a one sample *t*-test. To associate peaks with RNA-coding genes, BEDTools *closest* function was used to find TSSs from the RefSeq curated mRNA and non-coding RNA transcripts database located nearest to the center of each peak.

### DNA-binding motif analysis

Likely TRP47 DNA-binding motifs were identified using the MEME-ChIP tool in the Multiple EM Motif Elicitation (MEME) Suite [19,20]. BED files consisting of regions +/- 250 base pairs from the center of highly significant TRP47 binding peaks were generated using BEDTools. BED files consisting of only regions located within +/- 5 kb of associated gene TSSs according to GREAT were also generated. All BED files were converted to fasta format, randomly subsampled as needed to maximum 1000 sequences for manageable algorithm running time, and submitted to MEME-ChIP. MEME-ChIP executed complementary MEME and DREME algorithms to discover novel sequence motifs and CentriMo algorithm to measure positional enrichment of the *de novo* motifs and known motifs from JASPAR CORE vertebrates and UniPROBE mouse databases within peak sequences. Motifs that were statistically enriched in these regions were returned as probability matrices ranked by level of significance and grouped by similarity. Significant (*E-*value < 10^−5^) motifs were tested in multiple EMSAs.

### EMSA

Whole genomic DNA was isolated from healthy THP-1 cells and sheared using the ChIP-IT Express Enzymatic Kit (Active Motif) without crosslinking. Complete shearing of chromatin into fragments of 200 base pairs or less was verified by gel electrophoresis and ethidium bromide staining. Sheared genomic nucleic acid was then purified using the QIAquick PCR Purification Kit (Qiagen) and biotin labeled using the Label IT Nucleic Acid Labeling Kit (Mirus). EMSAs were performed using the Lightshift Chemiluminescent EMSA Kit (ThermoFisher). Briefly, 5 ng of labeled genomic DNA was incubated with up to 8 μg of purified protein in buffer containing 10 mM Tris, 50 mM KCl, 1 mM DTT, 5 mM MgCl_2_, 2.5% glycerol, 0.05% NP-40 and 25 ng/μl Poly (dI•dC). Samples were incubated at 4°C for 1 hr then separated on a 6% DNA retardation gel (ThermoFisher) at 100V for 90 min. Reactions were subsequently transferred to a Biodyne B nylon membrane (ThermoFisher) at 20V for 1 hr. Transferred DNA was crosslinked to the membrane using a CL-1000 Ultraviolet crosslinker (UVP) at 200 mJ/cm^2^ for 5 min. Reactions were imaged using streptavidin-HRP and x-ray film or a ChemiDoc-It^2^ Imager (UVP). EMSA reactions that included rabbit anti-TRP47 antibody at 1:200, 1:100, 1:50, and 1:20 dilutions were performed by adding antibody after 30 min of incubation and then incubating for an additional 30 min. Competition assays were performed using 500x molar increase of unlabeled DNA. Protein and unlabeled DNA were incubated for 30 min at 4°C followed by adding labeled DNA and incubating an additional 30 min. For EMSAs utilizing a probe, up to 2500 pM of labeled DNA was incubated with up to 10 μg of purified protein, 100-250x molar increase of unlabeled DNA, and 1:20 dilution of anti-TRP47 antibody.

Oligonucleotide probes (IDT) containing discovered TRP47 binding motifs were resuspended in annealing buffer (10 mM Tris, pH 7.5–8.0, 50 mM NaCl, and 1 mM EDTA) and complementary strands were annealed by heating to 95°C in a heat block for 5 min then slowly cooling to room temperature. Before use in EMSA, probes were diluted in annealing buffer. Sequences for probes P1, P2, P3, and P4 are listed in Table 1, and the discovered motifs are underlined.

**Table 1:** Probes used for TRP47 EMSA.

Probe	Sequence (forward strand, 5’→ 3’)
P1	TCAGGGCCTGGCTGCGCCTGCGCATCAG
P2	TCACGCACTGCGCGGCAGGGGCCGAGGCCTGTA
P3	CACCCTGCCCTGAGCCTGGGCTGGGGAGCA
P4	TCCGCCTCCCGGGTTCACGCCATTCTCCGTCTAA

## Results

### TRP47 localizes to the host cell nucleus in a mechanism involving a MYND-binding domain

**Fig 1:**
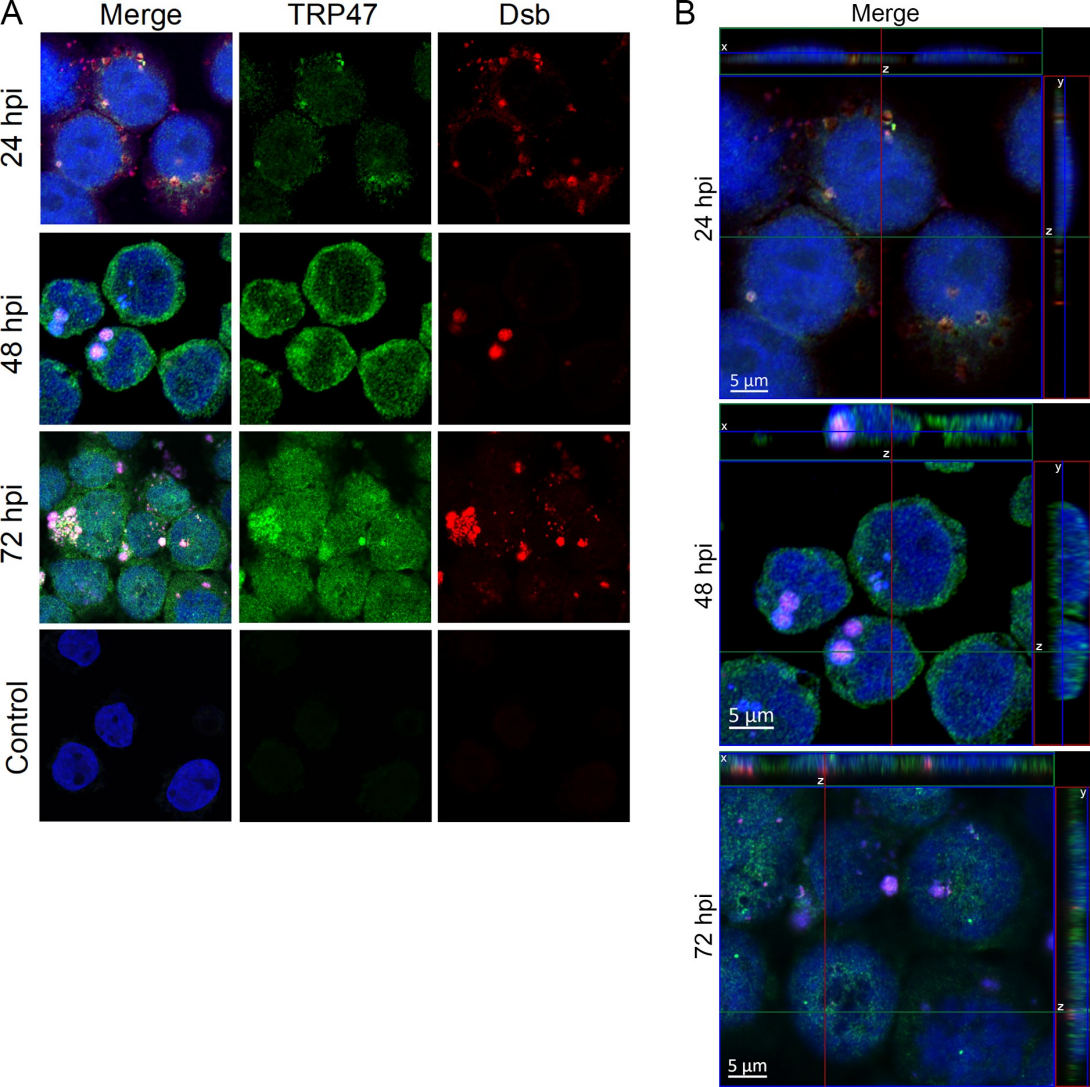
TRP47 localizes to the nucleus of *E*. *chaffeensis-*infected cells. (A) *E*. *chaffeensis-*infected and uninfected control THP-1 cells were fixed and probed with mouse anti-TRP47 (green), rabbit anti-Dsb (red), and DAPI (blue), and visualized by scanning confocal laser microscopy. For each timepoint post-infection, one representative optical slice from the *z* stack is shown. TRP47 was detected in all three timepoints in the infected cells. TRP47 was mainly found in cytoplasm at 24 hpi (in and around the morulae), whereas increased TRP47 was detected in the nucleus at 48 and 72 hpi. Ehrlichial Dsb (red) in micrographs confirmed presence of *E*. *chaffeensis* in infected cells at 24, 48 and 72 hpi. We did not observe staining for TRP47 or Dsb in uninfected control. (B) Orthogonal projections of optical slices from a z-stack confirmed the presence of TRP47 in the nucleus especially at 48 and 72 hpi. Top panels show an x-z projection and right panels show a y-z projection. The positions of the x and y axes within the projections denote the z depth of the slice shown in the center.

TRP47 nuclear localization during infection was examined using confocal microscopy (Fig 1). At 24 hpi, TRP47 is observed in and around morulae and around the nucleus of *E*. *chaffeensis-*infected THP-1 cells. At later timepoints 48 and 72 hpi, an increase in TRP47 was observed both in the cytoplasm and the nucleus.

**Fig 2:**
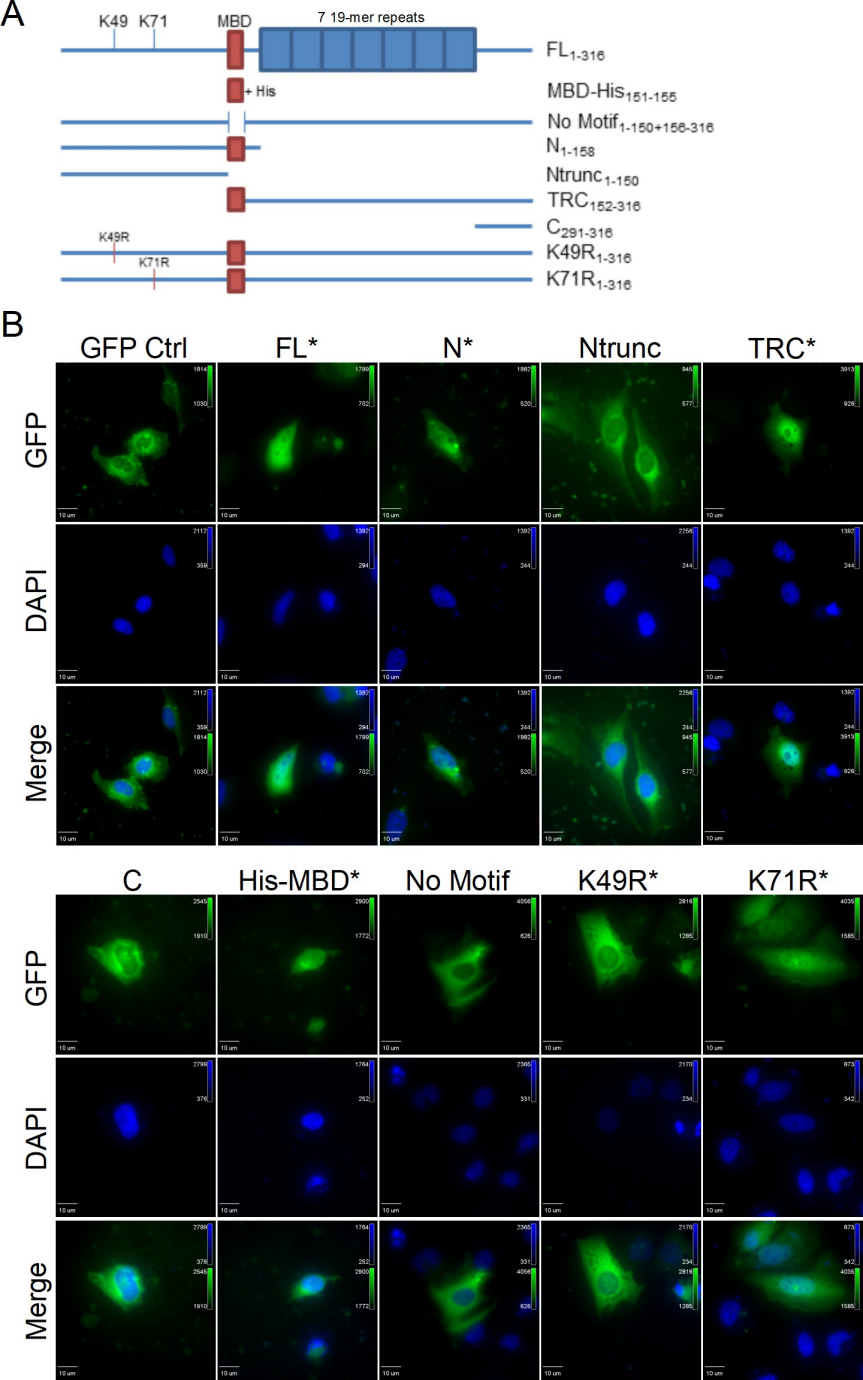
A MYND-binding domain is responsible for TRP47 nuclear translocation. (A) Schematic showing GFP-tagged TRP47 expression constructs. Red boxes indicate presence of the MYND-binding domain (MBD). Blue boxes represent the seven 19-mer repeats of the TRP47 tandem repeat region. Open space between two lines indicates a deletion mutation. Subscripts on construct labels indicate which amino acid residues are included. Constructs shown are full-length (FL), His-tagged MBD (MBD-His), full-length TRP47 without the MBD (No Motif), N-terminal (N), truncated N-terminal without the MBD (Ntrunc), tandem repeat-C-terminal overlapping the MBD (TRC), C-terminal (C), K49R mutant (K49R), and K71R mutant (K71R). (B) Ectopically expressed GFP-tagged constructs fluoresce green and DAPI-stained nuclei fluoresce blue. The FL, N, TRC, His-MBD, K49R, and K71R constructs contained the MBD (marked by asterisks), while the Ntrunc, C, and No Motif constructs did not. The FL and His-MBD TRP47 constructs exhibited strong nuclear localization and the N, TRC, and K71R TRP47 constructs exhibited nuclear and diffuse cytoplasmic localization. Exclusively cytoplasmic localization was observed with the Ntrunc, C, No Motif, and K49R mutant TRP47 constructs. The GFP negative control construct was also observed only in the cytoplasm. Due to differences in expression levels of the constructs, different exposure times were used to image each construct.

Analysis of the TRP47 amino acid sequence by the Eukaryotic Linear Motif resource identified a variant MYND-binding motif of PXLE (X: any amino acid) associated with binding of Hsp90 complex co-chaperones to PHD2 [21]. To determine the role of this domain in the subcellular localization of TRP47, a GFP control and TRP47 constructs encoding GFP-tagged full-length (FL), N-terminal (N), truncated N-terminal (Ntrunc), tandem repeat-C-terminal (TRC), C-terminal (C), His-MYND-binding domain (His-MBD), MBD deletion mutant (No Motif), K49R mutant, and K71R mutant TRP47 (Fig 2A) were ectopically expressed in HeLa cells and their localization observed by fluorescence microscopy (Fig 2B). All ectopically expressed TRP47 truncation constructs that included the MBD (marked by asterisks) except for K49R exhibited nuclear localization while constructs without the MBD exhibited exclusively cytoplasmic localization. These results indicate that the MYND-binding motif plays a role in TRP47 nuclear translocation. The inability of the K49R mutant TRP47 construct to enter or be retained in the nucleus could be due to a number of factors, such as unmasking of a nuclear export signal, loss of an important post-translational modification or protein-protein interaction, or both.

### TRP47 binds to host genomic DNA via a tandem repeat DNA-binding domain

**Fig 3:**
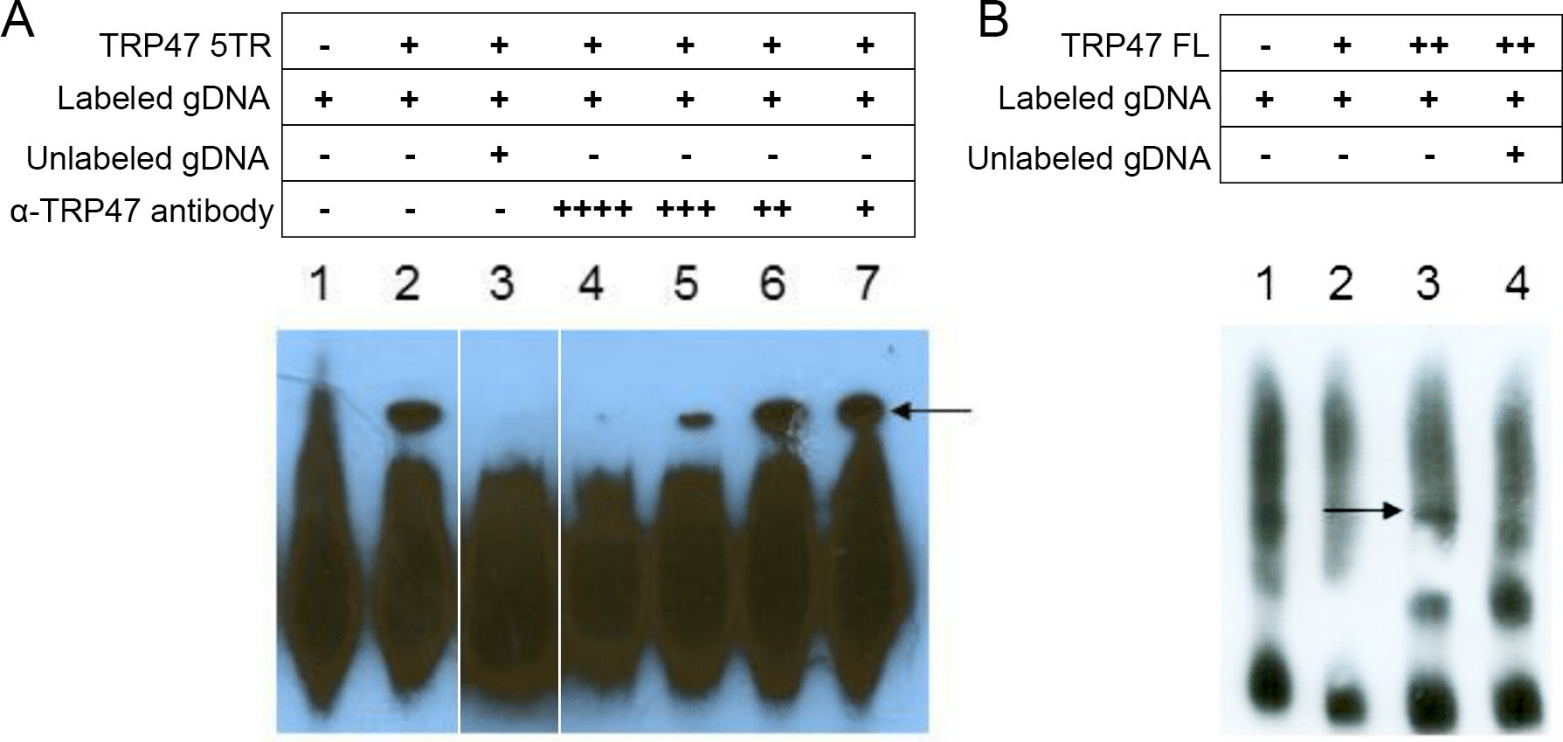
*E*. *chaffeensis* TRP47 binds host gDNA via the tandem repeat domain. (A) TRP47 5 tandem repeat (5TR) construct binds to host genomic DNA in EMSA. Incubation of biotin-labeled host gDNA with 4 μg TRP47 5TR (lane 2) yielded a strong shifted band (band height marked by arrow) compared to labeled gDNA alone (lane 1). Binding was prevented with addition of excess unlabeled competitor gDNA (lane 3), and with high concentration of anti-TRP47 antibody (lanes 4–7 are with 1:20, 1:50, 1:100, and 1:200, respectively). These lanes were rearranged from the original blot and white lines denote lanes that were not adjacent. (B) TRP47 full-length (FL) construct binds to host genomic DNA in EMSA. Incubation of labeled gDNA with 8 μg TRP47 FL (lane 3) yielded a shifted band (marked by arrow) not present with labeled gDNA alone (lane 1) nor with 4 μg TRP47 FL (lane 2). Binding was prevented with addition of excess unlabeled competitor gDNA (lane 4). A different gDNA preparation was used for the EMSA in panel B likely accounting for the difference in free gDNA migration and background.

To determine if TRP47 binds host genomic DNA, purified recombinant full-length and 5 tandem repeat (5TR) GST-tagged TRP47 constructs were used in an electrophoretic mobility shift assay (EMSA) with biotin-labeled sheared human genomic DNA. Signal detection in this assay is based on biotin-labeled DNA in complex with protein traveling more slowly during electrophoresis creating a shifted band. When an EMSA was performed with 4 μg of TRP47 5TR, formation of a protein-DNA complex was observed and addition of unlabeled competitor gDNA prevented detection of a band (Fig 3A), indicating that TRP47 specifically interacts with DNA. Addition of anti-TRP47 antibody resulted in loss of this band, most likely due to interference with the ability of TRP47 to bind DNA. Band intensity was gradually restored when antibody concentration was decreased, confirming that TRP47 is present in the protein-DNA complex. EMSA using full-length TRP47 showed formation of a protein-DNA complex that was not detected after the addition of unlabeled competitor gDNA (Fig 3B). These experiments confirm that TRP47 tandem repeat region is the functional domain for DNA binding. The amount of full-length protein required to visualize a band was twice the amount required for the tandem repeat construct, suggesting that full-length TRP47 has lower affinity for DNA than the tandem repeat region.

### TRP47 binds within regulatory regions of host genes involved in a variety of cellular processes

**Fig 4:**
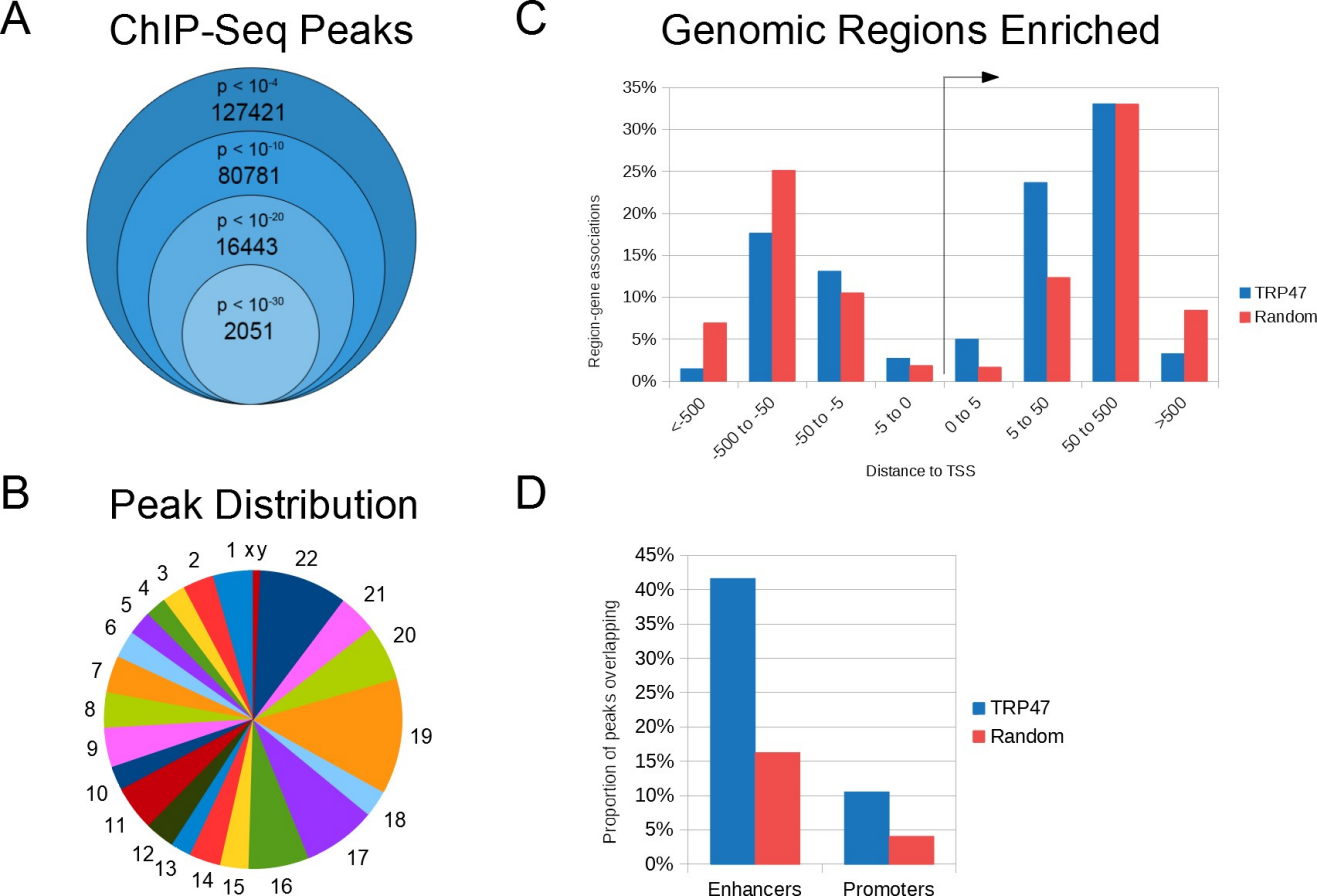
*E*. *chaffeensis* TRP47 binds at multiple locations in the host genome. (A) TRP47 chromatin immunoprecipitation resulted in significant enrichment of 127,421 regions (*p* < 10^−4^) compared to serum control and 2,051 peaks were highly significant (*p* < 10^−30^). (B) Number of TRP47-enriched regions per megabase of DNA for each chromosome. (C) TRP47 highly significant peaks were enriched within +/- 50 kb of host gene transcription start sites (TSS) compared to peaks randomly distributed in human genome. Some peaks were linked to more than one host gene and counted multiple times in this histogram. (D) TRP47 highly significant peaks were enriched in enhancer and promoter regions compared to random control peaks.

In order to identify TRP47 host genomic targets, ChIP-seq was performed. MACS identified 127,421 TRP47 binding peaks with *p* value < 10^−4^ and 2,051 highly significant peaks with *p* < 10^−30^ (Fig 4A). The enriched regions were distributed in all chromosomes (Fig 4B). Submitting highly significant peaks to GREAT revealed that the binding locations occurred at variable distances from the TSS of associated genes (Fig 4C). Compared to random control peaks, a significantly greater proportion of TRP47 peaks were located within +/- 50 kb (TRP47: 44.5%, random: 26.4%, *p <* 0.0001) and +/- 5 kb (TRP47: 7.7%, random: 3.5%, *p* < 0.0001) from TSS. Coverage analysis of binding locations using EPDnew promoter and GeneHancer enhancer region databases indicated that, compared to random control peaks, there was significantly greater enrichment of TRP47 peaks in promoters (TRP47: 10.6%, random: 4.1%, *p* < 0.0001) and especially enhancers (TRP47: 41.6%, random: 16.3%, *p* < 0.0001) (Fig 4D).

**Fig 5:**
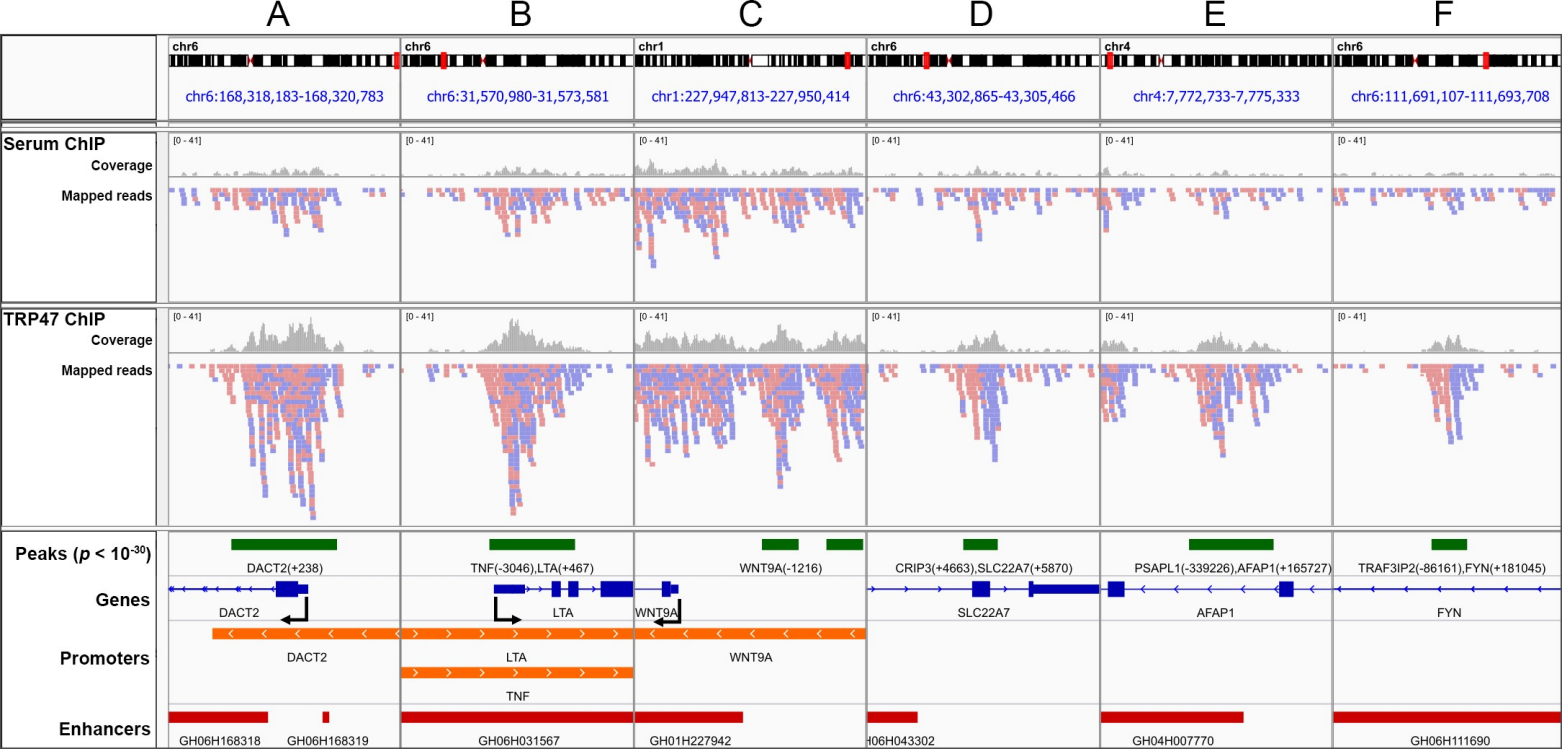
Visualization of *E*. *chaffeensis* TRP47 binding sites in genome browser. IGV was used to visualize TRP47 binding peaks in gene promoter and enhancer regions. Panels A-F represent genomic locations containing highly significant (*p* < 10^−30^) peaks. Serum and TRP47 ChIP tracks show forward (red) and reverse (blue) strand mapped reads and read coverage (gray bars). The tracks below depict peaks (green rectangles) labeled with associated genes and distance from TSS in parentheses; genes as exons (blue rectangles) and introns (blue lines) including 5' to 3' direction (blue arrows) and TSS (bent arrows); promoters (orange rectangles), specifically regions -5 kb to +1 kb from EPDnew primary promoter TSSs; and enhancers (red rectangles) labeled by GeneHancer ID number.

To assess the quality of the called TRP47 binding peaks, highly significant peaks were visualized with IGV (Fig 5). Panels A-C represent examples of peaks within promoters (A: DACT2, B: LTA and TNF, C: WNT9A), and panels E and F provide examples of peaks in enhancers (E: AFAP1, F: FYN). As expected for good quality ChIP-seq [22], the TRP47 ChIP sample forward and reverse reads are generally asymmetrically distributed around the midpoint of peaks and coverage within peaks is greater compared to serum control ChIP.

**Fig 6:**
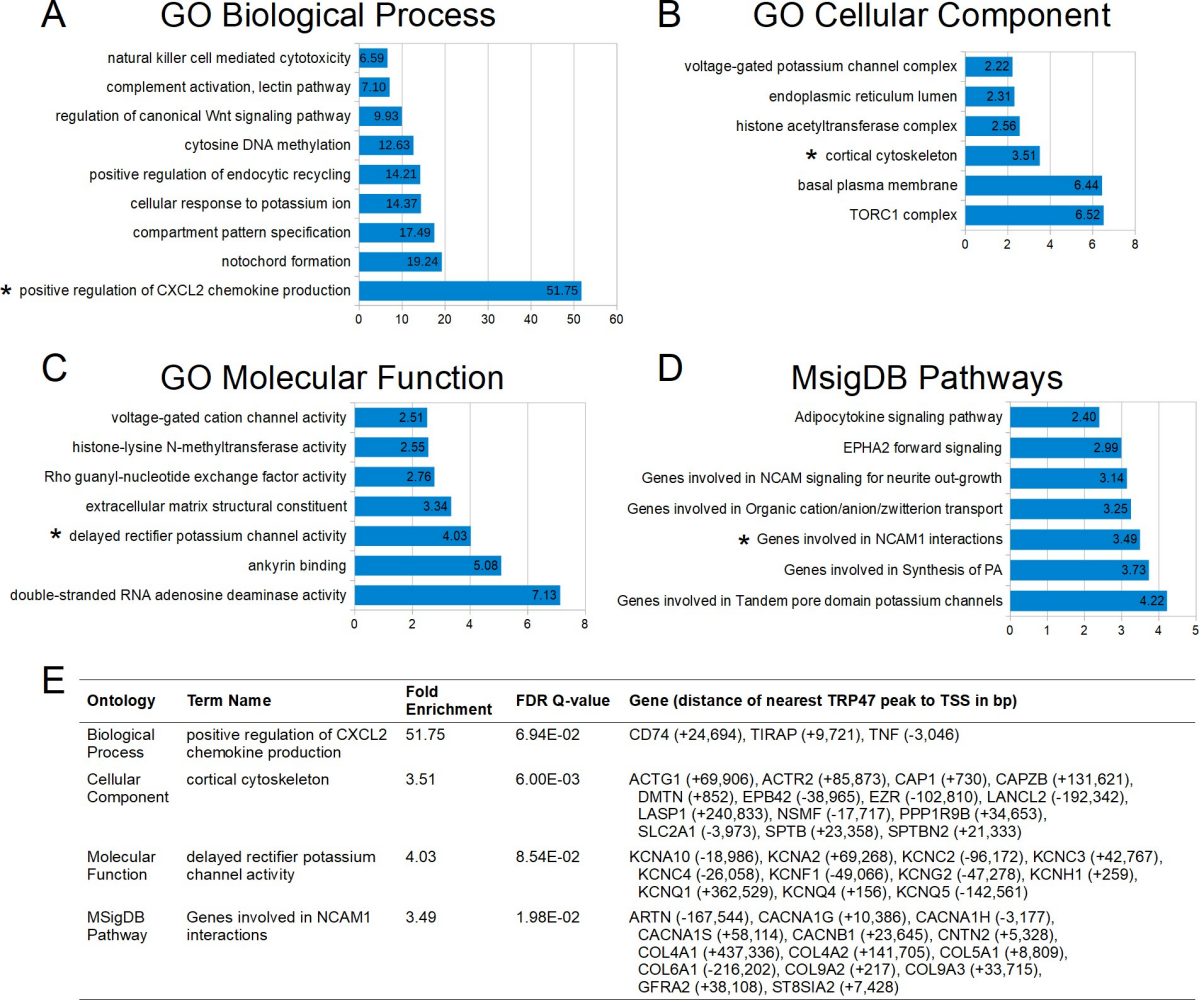
*E*. *chaffeensis* TRP47 targets include genes in several categories relevant to infection. (A) Selected Gene Ontology (GO) biological process terms that were significantly enriched by both region-based binomial and gene-based hypergeometric fold enrichment. All terms are presented by binomial fold enrichment. (B) Significantly enriched cellular component terms. (C) Significantly enriched molecular function terms. (D) Significantly enriched Molecular Signatures Database pathways terms. (E) A table showing selected enriched categories (marked by asterisks in panels A-D) with the corresponding binomial fold enrichment, hypergeometric FDR Q-value, and genes bound by TRP47.

**Fig 7:**
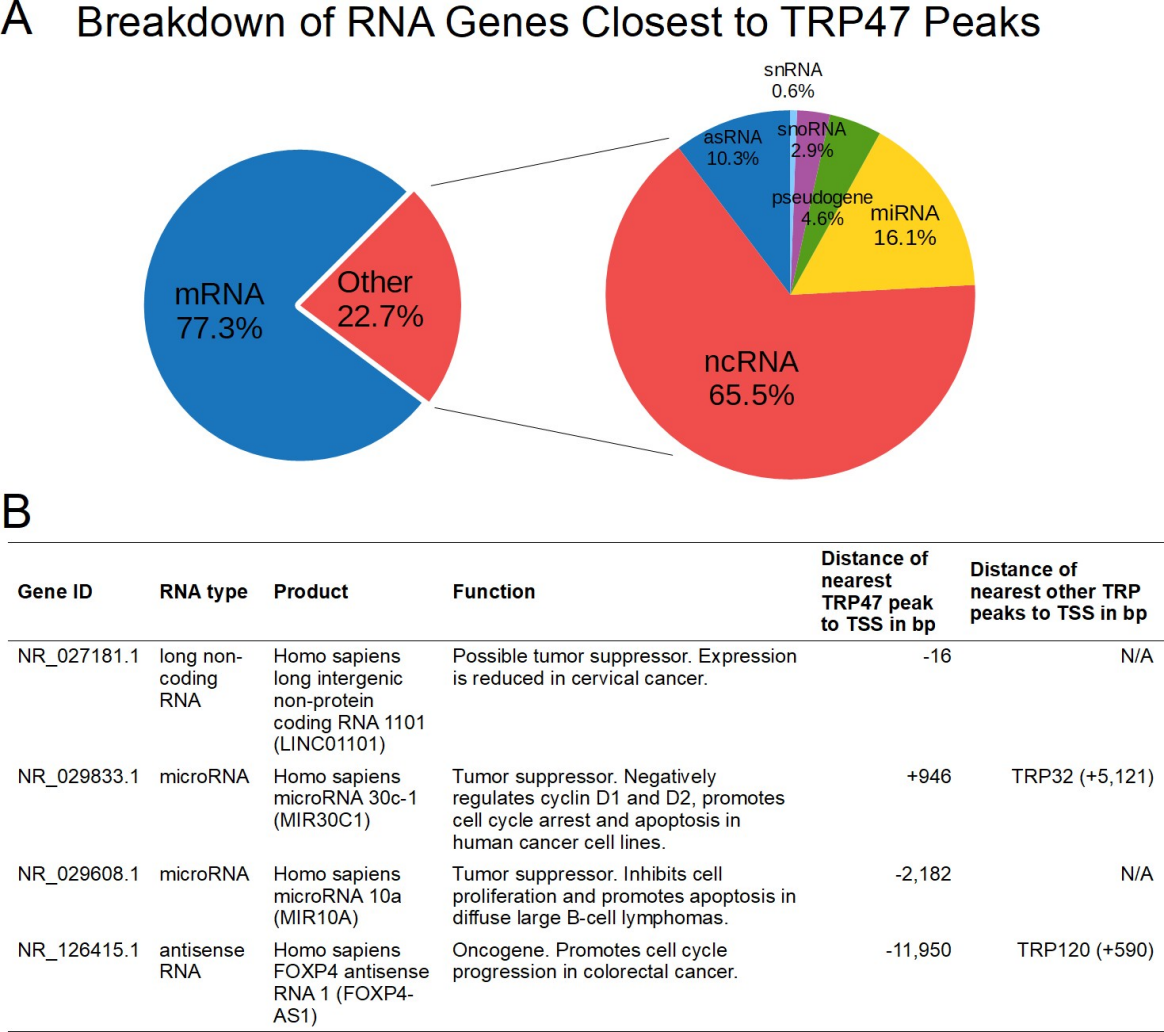
*E*. *chaffeensis* TRP47 binds to noncoding RNA genes in the host cell. (A) TRP47 targets include RNA-coding genes. When significant TRP47 binding peaks were submitted to BEDTools *closest*, 23% of peak-gene associations corresponded to RNA-coding genes. Of these, the majority were noncoding RNAs (66%) followed by microRNAs (16%), antisense RNAs (10%), pseudogenes (5%), small nucleolar RNAs (3%), and small nuclear RNAs (<1%). (B) A table of selected characterized noncoding RNA genes bound by TRP47 showing that many are involved cell proliferation or apoptosis and some are also bound by another ehrlichial TRP: NR_027181.1 [23], NR_029833.1 [24], NR_029608.1 [25], NR_126415.1 [26].

Using ontology term enrichment analysis, TRP47 binding regions were found to be associated with many functional categories of genes. The enriched categories within biological process, cellular compartment, molecular function, and pathways ontologies were related to innate immune response, cytoskeleton and vesicle trafficking, epigenetic regulation, cell differentiation, ion transport, RNA editing, and signal transduction including regulation of Wnt signaling (Fig 6A–6D). Multiple TRP47-bound genes were associated with each significantly-enriched category (Fig 6E). Further analysis of TRP47 binding sites revealed that most occur near protein-coding genes, but some are associated with RNA genes (Fig 7A). Of the TRP47-bound RNA genes that have a known or studied function, many have been implicated in various cancers and play a role in cell proliferation and apoptosis (Fig 7B).

### Multiple discovered TRP47 target genes and ontological categories overlap with those of TRP120 and TRP32

To examine potential interplay of ehrlichial nucleomodulins by targeting the same host genes or genes implicated in the same pathway or functional category, target genes and enriched ontology terms identified for TRP47, TRP32, and TRP120 were compared. For these comparisons, we used a different *p* value cutoff of peaks for each TRP to provide similar numbers of peaks. Significant terms in common between TRP47 and TRP120 (*n* = 48) corresponded to cellular processes such as intracellular transport, cell differentiation, and signal transduction (Table 2). Terms identified with more highly significant TRP binding sites (indicated by darker gray color) included regulation of protein kinase C and Notch signaling, endoplasmic reticulum lumen, and genes involved in NCAM1 (also known as CD56) interactions. Out of all genes associated with TRP binding peaks, 391 were in common for all three TRPs and 3,591 were in common for two TRPs. Among 4,703 genes associated with significant TRP47 peaks, 3,536 were in common with at least one other ehrlichial nucleomodulin while 1,167 were unique TRP47 targets. A subset of genes associated with highly significant TRP peaks are shown along with the TRP binding locations with respect to the TSS in Table 3. Categories of function of these genes included cell fate determination, transcriptional regulation, vesicle trafficking, cell signaling, and specifically Wnt signaling. Additionally, out of 1,323 previously identified Ank200 target genes [8], 557 were found to be in common with one or more TRPs. Lists of ontology terms and target genes identified for *E*. *chaffeensis* nucleomodulins are provided in S2 and S3 Tables, respectively.

**Table 2:** *E*. *chaffeensis* TRP47 and TRP120 common target genes.

Ontology Term	TRP120 peaks *p* value	TRP47 peaks *p* value
**GO Biological Process**		
CD4-positive or CD8-positive, alpha-beta T cell lineage commitment	1E-10	1E-25
dorsal/ventral neural tube patterning	1E-15	1E-30
positive regulation of protein kinase C signaling cascade	1E-20	1E-25
regulation of Notch signaling pathway	1E-15	1E-25
regulation of protein import into nucleus, translocation	1E-10	1E-30
vascular smooth muscle cell differentiation	1E-10	1E-25
**GO Cellular Component**		
anchoring collagen	1E-10	1E-25
cortical actin cytoskeleton	1E-10	1E-25
endoplasmic reticulum lumen	1E-15	1E-30
**GO Molecular Function**		
extracellular matrix structural constituent	1E-15	1E-30
**MSigDB Pathway**		
Adipocytokine signaling pathway	1E-10	1E-25
EPHA2 forward signaling	1E-10	1E-30
Genes involved in NCAM1 interactions	1E-20	1E-30
Regulation of RhoA activity	1E-10	1E-25

**Table 3:** *E*. *chaffeensis* TRP32, TRP47, and TRP120 common target genes.

Gene	Description	Categories of function	Distance of nearest TRP47 peak to TSS in bp	Distance of nearest TRP120 peak to TSS in bp	Distance of nearest TRP32 peak to TSS in bp
BMP8B	bone morphogenetic protein 8b	Cell differentiation, apoptosis	+12,250	-30,714	-56,920
CAP1	cyclase associated actin cytoskeleton regulatory protein 1	Actin cytoskeleton organization, signal transduction	+730	+395	+266
CDC20	cell division cycle 20	Anaphase promoting complex, cell proliferation, cell differentiation	+7,410	-9,967	+4,745
CITED4	Cbp/p300 interacting transactivator with Glu/Asp rich carboxy-terminal domain 4	Transcriptional regulation	-22,019	+552	+1,647
CLDN19	claudin 19	Cell-cell adhesion	-972	+4,212	+20,212
COL9A2	collagen type IX alpha 2 chain	Extracellular matrix	+1,489	+1,336	-5,285
CTPS1	CTP synthase 1	Metabolism, cell proliferation	+36,909	+38,366	+40,852
FOXJ3	forkhead box J3	Transcriptional regulation	+162,039	+163,774	+72,313
GUCA2A	guanylate cyclase activator 2A	Signal transduction	-105	-3,043	-35,933
IRF2BP2	interferon regulatory factor 2 binding protein 2	Transcriptional regulation, regulation of Wnt signaling pathway	-51,824	-497,747	-1,089
KCNQ4	potassium voltage-gated channel subfamily Q member 4	Potassium ion transmembrane transport	+156	+407	-21,608
LMNA	lamin A/C	Component of nuclear lamina, cell proliferation	+21,478	+20,220	+27,271
MFSD2A	major facilitator superfamily domain containing 2A	Phospholipid transmembrane transport	+1,243	+10,103	+17,522
MYCL	MYCL proto-oncogene, bHLH transcription factor	Transcriptional regulation, cell proliferation, cell differentiation, apoptosis	-20,520	+315	-15,102
NFYC	nuclear transcription factor Y subunit gamma	Transcriptional regulation	+66,525	+78,959	+42,829
PPT1	palmitoyl-protein thioesterase 1	Vesicle trafficking, metabolism	-34,059	+545	-5,957
PSMB1	proteasome subunit beta 1	Protein degradation, regulation of Wnt signaling pathway, transcriptional regulation, cell proliferation, cell differentiation	+42,531	+118,702	+114
RIMS3	regulating synaptic membrane exocytosis 3	Vesicle trafficking	-3,269	+16,041	+49,514
RLF	rearranged L-myc fusion	Transcriptional regulation	+226	-28,292	-3,314
TRIT1	tRNA isopentenyltransferase 1	tRNA modification, putative tumor suppressor	+130	+63,931	+11,964

To help validate host gene-TRP47 interactions, we checked if significant identified TRP47 target genes were found to be differentially expressed during *E*. *chaffeensis* infection in previous studies. Among 571 genes previously found to have significantly altered transcriptional levels for at least one time point post-infection [3], 102 are TRP47 targets and 21 of these are unique targets of TRP47 (S3 Table). Functional categories of these target genes include transcription, immune response, and apoptosis and cell proliferation. In addition, among 46 TRP32 target genes previously found to be differentially expressed during infection [10], 8 are also TRP47 targets, including CAP1, CMC1, HLX, ING1, MTFR2, NAT10, PARP16, and POLDIP3.

### TRP47 DNA motif

**Fig 8:**
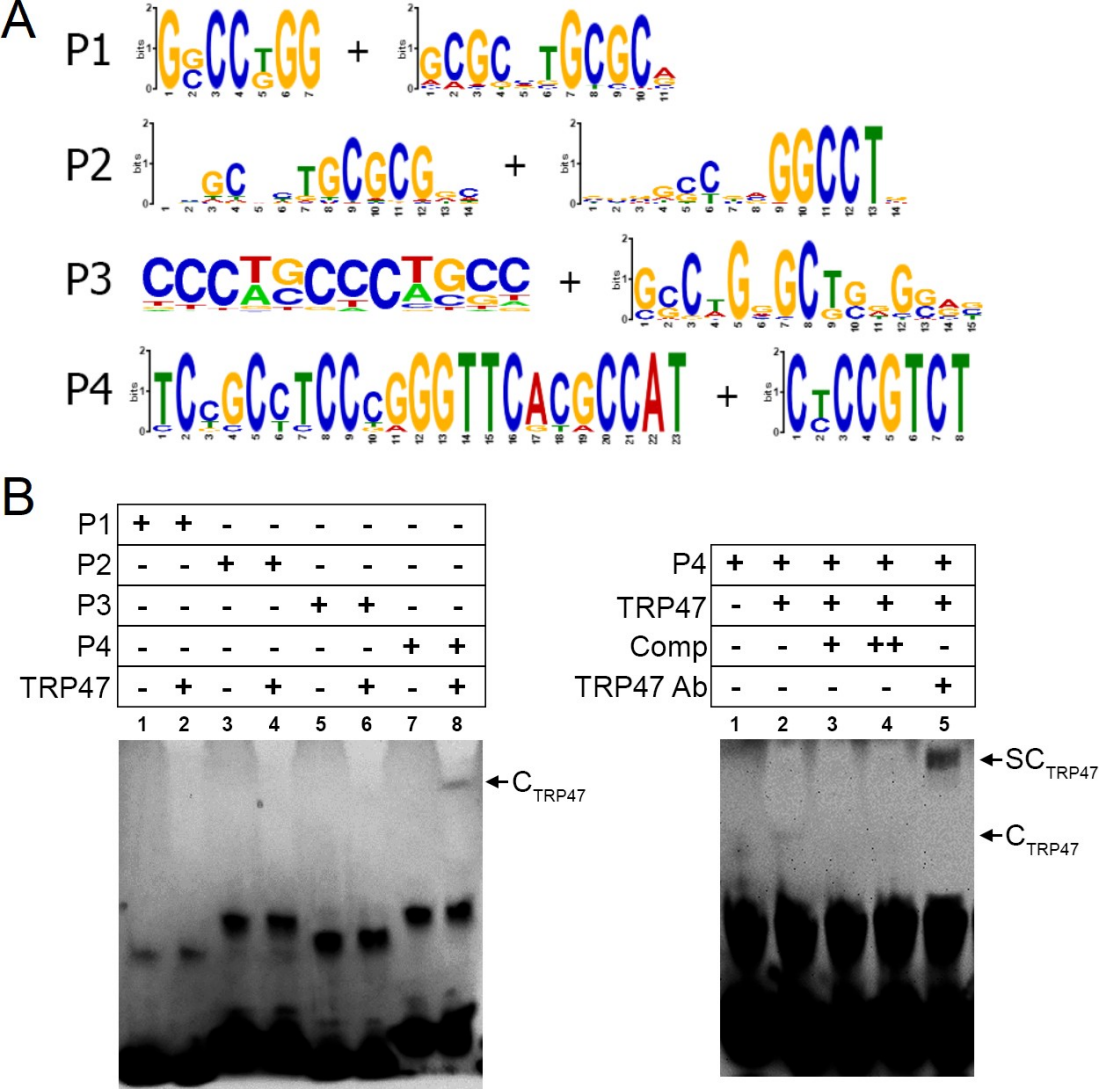
*E*. *chaffeensis* TRP47 binds a G+C-rich motif. (A) TRP47 was predicted to bind multiple G+C-rich motifs. The motifs incorporated into probes P1, P2, P3, and P4, (two motifs per probe, underlined in the probe sequences in Table 1) are shown in WebLogo format. (B) The predicted motifs were tested by EMSA. Incubation of biotin-labeled probes P1, P2, P3, and P4 with full-length TRP47 resulted in a shifted band complex C_TRP47_ for P4 (lane 8 in left panel and lane 2 in right panel, marked by arrows). The relative density of the shifted band was diminished with addition of 100 and 250-fold molar excess of unlabeled competitor DNA (right panel, lane 3 and 4 respectively). This band was also reduced with addition of anti-TRP47 antibody, but accompanied by a supershifted band complex SC_TRP47_ (lane 5, marked by arrow). These results suggest that P4 contains a specific TRP47 DNA-binding motif.

To determine the DNA motif bound by TRP47, highly significant ChIP peak sequences were entered into motif analysis software. Eight of the most likely TRP47 binding motifs were chosen as those with the lowest *E*-values returned from different sets of highly significant (*p* < 10^−20^) TRP47 peaks at any distance or exclusively within +/- 5 kb of gene TSSs (Fig 8A). To determine if the TRP47 ChIP-seq sites were bound directly by TRP47, we assayed the ability of purified TRP47 to bind candidate sequences *in vitro* using EMSA (Fig 8B). EMSA was performed using biotin-labeled probes (P1, P2, P3 and P4). TRP47 was not observed to bind P1, P2, or P3, but binding was observed for P4 (Fig 8B, left panel). Formation of the protein complex was inhibited by competition with excess unlabeled P4, and the complex was supershifted with addition of anti-TRP47 antibody (Fig 8B, right panel). We concluded that TRP47 specifically interacts with a conserved motif within P4. The specific TRP47 binding motif could be the consensus sequence [5’-CCTCCC-3’], which is only found within P4 and not P1, P2, or P3.

## Discussion

Survival and replication of *E*. *chaffeensis* and other obligately intracellular bacteria critically depends on subversion of host cell defenses and processes through diverse interactions with host molecules and pathways. An emerging mechanism utilized by intracellular pathogens to reprogram host cells involves secreted nucleomodulins that translocate into the nucleus and influence transcription. Recent studies demonstrated that *E*. *chaffeensis* effectors Ank200, TRP120, and TRP32 act as nucleomodulins that target genes involved in processes known or reasonably assumed to have an impact on ehrlichial survival. The results of this study suggest that TRP47 has similar nucleomodulin activity.

Previously, ehrlichial effectors Ank200, TRP120, and TRP32 were all found to localize to the nucleus of host cells during infection [8–10]. Nuclear localization for these effectors occurs later in the course of infection (after 48 hpi), although TRP120 was also detected in the nucleus as early as 3 hpi. Similarly, in this study, we observed that TRP47 is present in the nucleus mostly at 48–72 hpi with much less at 24 hpi. This suggests that TRP47 gradually accumulates in the nucleus over the course of infection. Although the TRPs lack a classical NLS, transport into the nucleus could involve post-translational modification and interaction with an NLS-containing partner (“piggy-backing”). For example, TRP32 nuclear localization requires phosphorylation of Y179 located in a PYYY motif, a post-translational modification that could directly or indirectly enable TRP32 to interact with a protein capable of trafficking to the nucleus. We found that the MYND-binding domain in TRP47 plays a role in its nuclear localization and that mutation of TRP47 K49 prevents nuclear localization. MYND domains mediate protein-protein interactions especially between transcription regulatory proteins. For example, the MYND domain in the ETO corepressor binds to the PPPL1 MBD in the SMRT and NCoR corepressors [27,28], and the MYND domain in the BS69 corepressor binds to the PXLXP MBD in the MGA transcription factor and viral transactivators E1A and EBNA2 [29]. The TRP47 MBD in TRP47, PXLE, is present in Hsp90 co-chaperones p23 and FKBP38 and binds the MYND domain of the HIF1-α hydroxylase PHD2 [21]. PHD2 has a non-classical NLS that is required for nuclear translocation [30]. The TRP47 MBD may allow binding to a MYND domain-containing protein such as PHD2 that directly or indirectly mediates TRP47 nuclear entry or retention. Although the precise mechanism by which TRP47 and other ehrlichial nucleomodulins access the nucleus remains to be elucidated, interactions with host proteins are likely involved.

The number of TRP47 ChIP-seq binding peaks called by MACS was greater than previously reported for TRP120 and TRP32. For instance, at the highly significant *p* value of 10^−20^, 16,443 TRP47 peaks were identified while 582 TRP120 and 241 TRP32 peaks were returned. These disparities could be accounted for by differences in the respective antibody specificity and its efficiency in immunoprecipitation or differences in technique such as chromatin fixation and sonication. The latter explanation may be supported by our observation of variable values of fold enrichment of DNA in positive control ChIP for different sheared chromatin preparations. There are also differences in the enrichment of binding peaks for each TRP within gene regulatory regions. While 20% of TRP120 and 43% of TRP32 highly significant peaks occur within +/- 5 kb of TSSs, 8% of TRP47 highly significant peaks occur at this distance. One possible explanation for the lower proportion of TRP47 peaks in promoter regions is that TRP47 preferentially binds enhancer regions to regulate host gene expression, which would represent a novel function of bacterial nucleomodulins. This hypothesis is supported by the fact that enrichment of TRP47 peaks is greater in enhancers (42%) compared to promoters (8%), and the degree of increased enrichment compared to random peaks is greater in enhancers (+25.3%) compared to promoters (+6.5%).

Enriched ontological categories and even gene targets identified for TRP47 alone or in addition to TRP32 or TRP120 included many that have been previously reported to be exploited by *E*. *chaffeensis* to facilitate infection. For example, TRP32, TRP47, and TRP120 all interact with proteins involved in Wnt signaling, TRP120 interacts with Notch metalloprotease ADAM17, and both pathways are required for ehrlichial survival [31,32]. Correspondingly, among the significant TRP47 target gene processes identified in this study were the Notch signaling pathway, which was also implicated with TRP120 peaks, and regulation of canonical Wnt signaling. TRP47-bound genes involved in these pathways were identified as well, including PSMB1 and IRF2BP2, both of which were also identified as TRP32 and TRP120 target genes. IRF2BP2 also encodes a host protein target of TRP120, suggesting it is regulated in some way by TRPs both at the level of transcription and protein. It is possible that *E*. *chaffeensis* utilizes multiple effectors to regulate expression of the same host target genes in order to ensure effective regulation of genes critical for its survival.

Many of the identified TRP47 target genes have been found to be differentially expressed during *E*. *chaffeensis* infection in previous studies [3,10]. This suggests that TRP47 may play a key role in regulating expression of the identified target genes. However other factors could be at least partially responsible for altered expression of the TRP47-bound genes. For instance, 81 of 102 differentially expressed TRP47-bound genes are also identified targets of other ehrlichial nucleomodulins; TRP47 may or may not be the primary nucleomodulin responsible for altering expression of these genes. Further, other processes related to infection can modulate gene transcription, such as effector-mediated modulation of cell signaling and host cell immune response.

TRP47 was found to bind host DNA via its tandem repeat domains, similar to TRP120 and TRP32 in *E*. *chaffeensis* and the TAL family of effectors in *Xanthomonas*. The same amount of full-length TRP47 produced a much weaker band shift than the tandem repeat construct. This comparatively reduced affinity of full-length TRP47 for DNA could be due to the presence of an inhibitory domain in the N-terminal or C-terminal portion of the protein. In the supershift assay, a gradual inhibition of DNA-binding activity occurred with increasing concentration of antibody. This phenomenon has been previously described for several transcription factors [33,34] and is likely due to competition between the antibody and DNA binding of the TRP47 tandem repeat region since the antibody used was directed against a molecularly defined epitope in the tandem repeat domain. Interestingly, a recent study using nuclear magnetic resonance spectroscopy found that TRP120-1TR construct specifically binds DNA only at low pH and with affinity in the micromolar range weaker than that of zinc finger transcription factors [35]. Further, TRPs may require a post-translational modification or interaction with host or ehrlichial proteins to adopt an ideal conformation for DNA binding, neither of which can occur in the *in vitro* EMSA assay. These features of ehrlichial TRP-DNA binding may account for difficulties with acquiring strong shifted bands in TRP47 EMSAs with gDNA and synthesized probes.

MEME-ChIP analysis using TRP47 peaks returned many statistically significant candidate TRP47 binding motifs that were rich in guanine and cytosine nucleobases, similar to TRP120 and TRP32 motifs. Compared to the shifted bands seen in EMSA with TRP47 and gDNA as well as the other TRPs and their respective positive probes, the shifted bands we observed in multiple EMSAs using probe P4 with TRP47 were weaker in intensity. One possible reason for the relatively low binding affinity for this probe is that only a single iteration of the TRP47 motif is present and a repeat is required for strong protein-DNA interaction. This is supported by the fact that both the TRP120 and TRP32 probes that demonstrated strongest interactions in EMSA had two 6-nucleotide inverted repeats in close proximation, a feature that has also been reported in DNA-binding sites of other transcription factors such as EBF [36]. The positive EMSA result for probes containing the discovered TRP47 motifs helps validate the ChIP-seq peaks and affirm that they represent true TRP47 binding sites.

In this study, we identified an additional ehrlichial effector that is likely involved in direct transcriptional regulation of host genes relevant to infection. We demonstrated that TRP47 translocation to the nucleus involves a MYND domain, where it binds host DNA, and host targets include genes involved in signal transduction, vesicle trafficking, and immune response. Further research will evaluate direct modulation of expression of target genes by TRP47 and elucidate the mechanisms and pathological significance of regulation of host genes by ehrlichial nucleomodulins.

## Supporting information

S1 Table Oligonucleotide primers used to create *E*. *chaffeensis* TRP47 expression constructs. (DOC)

S2 Table Ontology terms identified for TRP47, TRP32, and TRP120. Significantly enriched terms within the GO Biological Process, GO Cellular Component, GO Molecular Function, MSigDB Pathway, and PANTHER Pathway ontologies identified by GREAT for each TRP are presented. In the TRP columns, the lowest *p* value peak set which returned the term association is shown. The *p* value cutoff used varied by TRP to provide similar numbers of peaks: p < 10^−25^ for TRP47, p < 10^−4^ for TRP32, and p < 10^−10^ for TRP120. Enriched gene ontology terms for Ank200 were reported previously [8].
(XLSX)

S3 Table Target genes identified for TRP47, TRP32, TRP120, and Ank200. Genes that were identified as significant targets for at least one *E*. *chaffeensis* nucleomodulin are listed. In each TRP column, the lowest *p* value peak nearest to the associated gene TSS and the distance to TSS is shown. In the Ank200 column, the FDR value of the previously identified gene interaction [8] is shown. In the Induced or Repressed column, results from a previous study of differential gene expression during *E*. *chaffeensis* infection [3] are shown.
(XLSX)
