# Supplementary material for: Ehrlichia chaffeensis TRP47 enters the nucleus via a MYND-binding domain-dependent mechanism and predominantly binds enhancers of host genes associated with signal transduction, cytoskeletal organization, and immune response
Source: PLoS One. 2018 Nov 8;13(11):e0205983. doi: 10.1371/journal.pone.0205983 (PMC6224051; doi:10.1371/journal.pone.0205983)
Supplement: S1 Table — (DOC) [file pone.0205983.s001.doc]

**Table S1. Oligonucleotide primers used to create *E. chaffeensis* TRP47 expression constructs.**

| **Target^a^** | **Forward Oligonucleotide Sequence** | **Reverse Oligonucleotide Sequence** | **Product Size (bp)** |
| --- | --- | --- | --- |
| **GST constructs** | | | |
| FL_1-316_ | aaaagaattcatgcttcatttaacaacag | aaaagtcgacgaaataaaagtatctattacc | 968 |
| 5TR_159-253_ | aaaagaattcgctagtgtatctgaaggag | aaaagtcgactgcaggagtttcttggctt | 381 |
| **GFP constructs** | | | |
| FL_1-316_ | aaggcctctgtcgacatgcttcatttaacaacagaaatt | agaattcgcaagcttttagaaataaaagtatctattaccaa | 981 |
| N_1-158_ | aaggcctctgtcgacatgcttcatttaacaacagaaatt | agaattcgcaagcttatttccttcaagaactggaac | 501 |
| Ntrunc_1-150_ | aaggcctctgtcgacatgcttcatttaacaacagaaatt | agaattcgcaagcttcactatactgtcacttaaagat | 477 |
| TRC_152-316_ | aaggcctctgtcgacggaaatgctagtgtatctgaa | agaattcgcaagcttttagaaataaaagtatctattaccaa | 495 |
| C_291-316_ | aaggcctctgtcgacactcaaccacaatctagagat | agaattcgcaagcttttagaaataaaagtatctattaccaa | 111 |
| His-MBD_151-155_ | aaggcctctgtcgacgttccagttcttgaacaccaccaccaccaccac | agaattcgcaagcttgtggtggtggtggtggtgttcaagaactggaac | 48 |
| **Mutagenesis** | | | |
| K49R | ggaagtgaacctgatcatggttatcatattttatttagaaacaatggtcatgttatat | atataacatgaccattgtttctaaataaaatatgataaccatgatcaggttcacttcc | - |
| K71R | ggtgtacaagctgaaaactttgtatttgatataagaaatcacaatttaagagct | agctcttaaattgtgatttcttatatcaaatacaaagttttcagcttgtacacc | - |

^a^Subscripts represent the amino acids contained in the fragment
